# Growing Up in the Great Recession: The Effects of Three Dimensions of Economic Well-being on Child Behavioral Difficulties from Ages 3 to 17

**DOI:** 10.1007/s10964-022-01721-2

**Published:** 2023-01-13

**Authors:** Ryan Alberto Gibbons, Stefanie Sprong, Yekaterina Chzhen

**Affiliations:** Department of Sociology, Trinity College Dublin, The University of Dublin, Dublin, Ireland

**Keywords:** Growing up in Ireland (GUI), Financial strain, Material deprivation, Household income, Child behavioral difficulties, Fixed effects analysis

## Abstract

Empirical research into the relationship between economic well-being and child outcomes has been limited by its cross-sectional nature, or its narrow focus on predominantly financial aspects of economic well-being. This article attempts to overcome these shortcomings by using data from the Growing Up in Ireland Cohort98 (age: 9–17; N = 5,748; female: 51.4%) and Cohort08 studies (age: 3–9 years; N = 7,208; female: 49.8%), which cover a period of large macroeconomic fluctuation (2007–2017). This fluctuation makes a robust fixed effects analysis feasible, allowing for economic well-being effects to be isolated by controlling for all time-invariant confounders. The article uses three different measures of economic well-being (subjective financial strain, material deprivation, income) to explore how distinct forms of economic well-being affect child behavior. The results suggest that household income is not related to behavioral difficulties, whereas subjective financial strain is predictive of externalized behavioral difficulties in adolescent boys. Material deprivation is predictive of externalized behavioral difficulties in adolescent boys and internalized behavioral difficulties in younger boys, but has no effect on girls’ behavioral outcomes. The findings indicate that the relationship between economic well-being and child behavioral outcomes is complex, and requires multi-dimensional measures of economic well-being to accurately ascertain the different effects.

## Introduction

Researchers have long been interested in the effects of economic well-being on children’s development, with many studies reporting the deleterious effects of experiencing economic hardship during childhood (c.f. Cooper & Stewart, 2021). However, fewer studies have looked at the heterogeneous effects of the different dimensions of economic well-being, with a few notable exceptions (e.g. Schenck-Fontaine & Panico, 2019). Moreover, most do so cross-sectionally (e.g. Bradshaw & Finch, 2003), thereby failing to identify how different aspects of economic well-being may develop over time and affect child outcomes over the early life course. This is unfortunate because taking such a view would offer a more comprehensive understanding of the effects of economic hardship on children. This study sets out to reach a more robust understanding of how different experiences of economic hardship affect children’s behavior by disentangling the effects of three separate dimensions of economic well-being, and evaluating their effect on child internalized (e.g. psychological and somatic) and externalized (e.g. conduct and hyperactivity) behavioral difficulties. This is made possible through the use of data from three waves of Cohort98 (GUI98) and Cohort08 (GUI08) studies of Growing Up in Ireland, two nationally representative longitudinal datasets of children in Ireland that coincided with a period of major society-wide economic contraction. Utilizing this variation in economic well-being over time to better isolate their effect on child behavioral problems, the article attempts to answer the following research question: to what extent are the different dimensions of economic well-being (i.e., disposable household income, material deprivation, and financial strain) associated with internalized and externalized behavioral problems in middle childhood and early adolescence?

### The Effect of Household Economic Well-being on Children

Household economic well-being has long been identified to hold a strong association with mental, cognitive and physiological outcomes in children. Children growing up in households reporting economic hardship are, for instance, more likely to perform worse in school (Layte, 2017), to report poorer life satisfaction and worse mental health (Chzhen et al., 2021), to have fewer friends and experience greater social isolation (Hjalmarsson and Mood, 2015), and to engage in more deleterious and risky behavior (Kaiser et al., 2017) relative to peers growing up in households experiencing more favorable economic circumstances. Furthermore, such repercussions do not only affect the child at that point in time, but also hold negative consequences for later life (Cooper & Stewart, 2021; Van Lancker & Vinck, 2019; Whitfield et al., 2021).

The significant implications of an association between experiences of economic hardship in a household and repercussions for children has led to the development of theoretical frameworks that seek to explain the mechanism behind the relationship. The Family Investment Model (FIM), for instance, hypothesizes that the association reflects parental financial, social and human investment in the child (Conger & Donnellan, 2007). Meanwhile, an alternative Family Stress Model (FSM) argues that the effect of household financial strain and the negative consequences this has on interparental and parent-child relationships mediates the association between economic hardship and child outcomes (Conger, 1994). Yet, while both provide strong theoretical and empirically tested models, they are arguably limited by a focus on specific forms of economic well-being. FIM, for instance, primarily focuses on family resources such as income and the role they play in the purchase of material and immaterial child benefits, and FSM, while focusing on the role of financial stress, views it as a product of low or unstable income and not as a separate experience of economic well-being itself.

The narrow focus of these studies raises questions as to whether the models fully capture the mechanisms between different experiences of economic hardship, with it being increasingly recognized that household-based monetary metrics do not fully explain what it means for a child to grow up in a poor household (Chzhen et al., 2018). Children in households above the national monetary poverty line may still be deprived in key dimensions of child well-being (Chzhen & Ferrone, 2017), while some children in income-poor households may be shielded from deprivation (Main & Bradshaw, 2016; Sprong et al., 2022). Moreover, there is a temporal dimension to the relationship between different domains of economic hardship. Nolan et al. (2006) and Nolan and Whelan (1996) argued that the limited overlap between income poverty and material deprivation observed in Irish living conditions data in the 1990s was because it takes time for consistently low income to translate into inability to afford socially perceived necessities (i.e. material deprivation) and for rising income to result in improved living conditions. This suggests that changes in different dimensions in economic well-being may affect children differently. Parents may find it easier to hide temporary income losses from their children (e.g., by using savings or credit) than to shield them from increasing difficulties to make ends meet or to afford socially perceived necessities (e.g., due to increasing costs of living).

This has therefore prompted research to adopt more nuanced interpretations of economic well-being, looking at combinations of household disposable income, deprivation and reported financial strain to ascertain a more complete picture of a household’s economic well-being (Bradshaw & Finch, 2003). However, such studies have themselves been limited by a reliance on predominantly cross-sectional analysis, constraining their ability to identify causal effects. Even in studies that have adopted longitudinal approaches, such as Schenck-Fontaine and Panico (2019), the ability to identify distinct effects have been limited owing to the stability of measures within households over time, making disentangling the effects of economic well-being variables from potential confounders difficult.

Thus, it remains an open question whether all experiences of economic hardship have similar effects on the development of child outcomes, and whether dimensions of economic hardship hold independent effects even when experienced simultaneously with other dimensions. In particular, gaining a better understanding of whether distinct dimensions of economic hardship can affect the development of child behavioral difficulties differently is important, given the deleterious effect child behavioral difficulties have been observed to hold on numerous child outcomes (Plenty et al., 2021).

### Associations with Behavioral Difficulties

Child behavioral difficulties are a key area of interest for researchers exploring the effect of economic hardship on children, both on account of behavioral difficulties being disproportionately present among children exposed to economic hardship in the household, and its association with other negative outcomes, such as poor student performance net of the child’s ability, family background and sex (Layte, 2017). The association between socio-economic background and child behavioral difficulties has been identified in multiple studies, with children from lower socio-economic backgrounds being observed to exhibit behavioral difficulties more frequently than their socio-economically advantaged peers (Flouri et al., 2015; Reiss, 2013). Furthermore, children growing up in poor neighborhoods appear to develop behavioral difficulties more readily than their peers in more middle class neighborhoods, although effects appear to be conditional on factors such as race/ethnicity and sex (e.g. Clampet-Lundquist et al., 2011). This indicates that more affluent social surroundings can potentially act as buffer against the development of behavioral difficulties, even when exposed to the same risk factors (Schonberg & Shaw, 2007).

However, although correlated with socio-economic background, the mechanism behind the development of behavioral difficulties appears to be driven primarily by exposure to economic difficulties. This relationship between exposure to economic hardship at the household level and child behavioral difficulties has been predominantly theoretically mechanized through the FSM, whereby financial stress negatively affects familial processes and relationships, leading to a deterioration in the quality of parent-child relationships and the emergence of child behavioral difficulties (Conger & Conger, 2002). A large literature has tested these FSM pathways and introduced others (see Masarik & Conger, 2017), such as adolescent coping mechanisms mediating the effects of family financial stress on adolescent mental health (Wadsworth & Berger, 2006).

Such models focus on the experience of financial strain, itself predicted by income and material deprivation. Yet, given that different dimensions of economic hardship are not always experienced together and can occur independently (Roelen & Notten, 2013), it is worth investigating if these measures may also have different independent effects on the development of child behavioral difficulties. For instance, a child in a family that is experiencing financial strain despite not being income poor might still be expected to exhibit behavioral difficulties in line with the FSM hypothesis, yet a child in a household that has experienced a drop in income and lives below the poverty line but does not feel financially strained might not develop behavioral difficulties. Factors such as household debt and savings might also drive experiences of financial strain and material deprivation yet be missed with conventional measures of household income (Headey, 2008). Similarly, a household may also experience strain, but the absence of income poverty or material deprivation may buffer the child from the development of behavioral difficulties.

It is also important to understand if changes in different dimensions of household economic well-being have different implications for younger and older children. It is well documented that household resources play a key role in children’s early development (Duncan et al., 2010; Longo et al., 2017), while conversely family resources have been observed to be less associated with the development of internalized behavioral outcomes among older children as youth’s own economy takes on increasing relevance (Plenty & Mood, 2016). Meanwhile, a rich body of evidence from the international Health Behavior in School-age Children surveys shows that poor subjective well-being and psychosomatic health complaints are more prevalent in adolescents from less well-off households (Elgar et al., 2015). While much of the FSM literature focuses on adolescents, especially in the earlier iterations of the model (Barnett, 2008; Masarik & Conger, 2017), the FIM literature emphasizes the importance of the preschool and early school years (Yeung et al., 2002). Although the early years are a key developmental period for cognitive and social skills acquisition (see Knudsen et al., 2006), adolescence is increasingly viewed as a “second window of opportunity” for prevention and early intervention and a sensitive period for the effects of stress on mental health (Fuhrmann et al., 2015). Yet the authors of this article are not aware of studies comparing the effects of different dimensions of household well-being on behavior problems in both young children and adolescents.

### Irish Context

Seminal studies exploring the effect of changing economic well-being on family processes and child outcomes have often been produced in the aftermath of sustained and severe economic contraction, such as the Great Depression (Elder, 1974), the Farm Belt Depression in the American Midwest (Conger et al., 1994), and the economic recession in Finland in the early 1990s (Solantaus et al., 2004). The Great Recession in Ireland provides a similarly relevant case-study for the 21^st^ century. The country moved from an environment of sustained economic growth, negligible unemployment and net immigration in the years immediately prior to the global economic downturn, to a situation of severe economic contraction and austerity, 15% unemployment, net emigration and a financial bailout at its height (Eurostat, 2021). While the recession led to a heterogeneity of experiences (Maître et al., 2021; Sprong et al., 2022), few households were left untouched by the recession, with observational studies identifying that the majority of households experienced significant recessionary effects (Reinhard et al., 2018; Watson et al., 2016) and an average drop in household income of over 17% (Layte & McCrory, 2018).

## Current Study

Previous research has identified an association between economic well-being and children’s internalized and externalized behavior, yet research investigating whether different experiences of economic well-being affect child behavioral development in distinct ways is more limited. The paucity of previous theory and research evaluating different effects from distinct measures of economic well-being makes the development of hypotheses difficult. However, the well documented association between economic well-being and child behavior lead us to expect that each of the three dimensions of economic well-being (income, material deprivation and financial strain) will be associated with child behavior, with declining economic well-being in each of the measures associated with worsening child behavior (Hypothesis 1). Furthermore, it is anticipated that the effect of economic well-being on child behavioral outcomes will manifest itself differently in different age cohorts. The behavioral outcomes of younger children will be more readily affected by well-being measures such as income and material deprivation that are associated with household resources (FIM), while financial strain and the psycho-social implications associated with it will have a more defined effect on the behavioral outcomes of adolescent children (FSM) (Hypothesis 2).

## Method

### Data

The analyses utilize two longitudinal datasets of children and young people in Ireland: GUI98 and GUI08. GUI98 and GUI08 are nationally representative datasets of children in the Republic of Ireland born in 1998 and 2008 respectively, and of their households. Data for Wave 1 of GUI98 was collected from the end of 2007 to the start of 2008 when the children were nine years old (N = 8,568), for Wave 2 from the end of 2011 to the start of 2012 when the children were 13 years old (N = 7,525), and between 2015 and 2016 for Wave 3 when children 17/18 years old (N = 6,216). Data for Wave 2 of GUI08 was collected between the end of 2010 and early 2011 when children were 3 years old (N = 9,973), mid 2013 for Wave 3 when children were 5 years old (N = 9,001), and from mid-2017 to early 2018 for Wave 5 when children were 9 years old (N = 8,032). Two additional waves of data from GUI08, Wave 1 when children were 9 months old (2007/8), and a postal survey at Wave 4 when children were 7/8 years old (Spring 2016), do not contain the outcome measures and are therefore not included in the analyses.

For GUI98, the sample was drawn from approximately 56,500 nine year-olds registered in the 2006 census, with the sample size at Wave 1 of 8,568 participants representing approximately one in every seven nine-year-old child residing in the Republic of Ireland at the time (Thornton et al., 2016). Children and their families were selected for inclusion in the study following a two-stage process, whereby first schools were selected, with children being selected from within schools in the second stage. With the majority of schools in Ireland having fewer than 40 nine-year-old students, increasing the probability of a larger school being selected was necessary in order to correctly collate a representative sample. As such, in the first stage primary schools with fewer than 40 nine-year-old students were selected on a systematic stratified basis (geographic location, disadvantaged status, religious denomination, categorical size and gender mix). For schools with greater than 40 nine-year-old students, schools were selected on a probability proportionate to size approach, giving larger schools a greater probability of selection. In the second stage, the increased probability of larger schools being selected was counterbalanced by the probability of a student being selected being negatively associated with the number of students in the school, whereas all nine-year-old students from schools with fewer than 40 nine-year-old students were eligible for selection. For GUI08, infants were selected from the national Child Benefit Register so as to be 9 months of age at the time of interview (between September 2008 and April 2009), yielding a total eligible population of 41,185 children. The sample was then selected from the eligible population using a systematic approach, pre-stratifying by maternal marital status, county of residence, nationality and number of children – variables accessible from the Child Benefit Register. Infants were then randomly selected from the stratified samples (Williams et al., 2009).

**Fig. 1:**
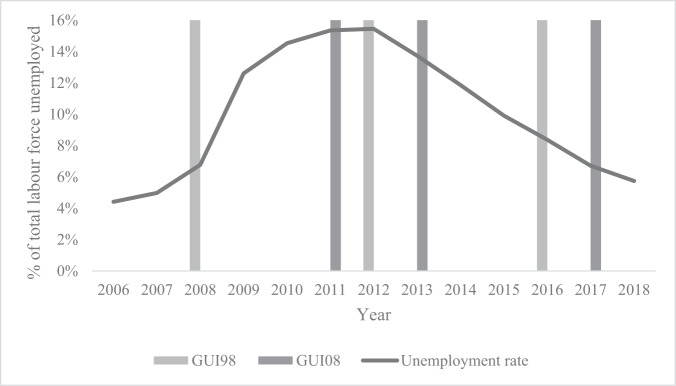
Unemployment rate (% of total labor force) in the Republic of Ireland, overlapped on the GUI98 and GUI08 data collection timepoints. Note. In instances where data collection took place over two calendar years, the year in which the majority of data collection took place is indicated. Unemployment statistics obtained from Central Statistics Office (Central Statistics Office, 2021a)

In both GUI98 and GUI08, interviews were conducted with children and their parent(s)/guardian(s) at home. Parent(s)/guardian(s) were self-identified as primary and (where relevant) secondary caregivers, with the study child’s mother (biological or otherwise) being self-identified as the primary caregiver in nearly all instances. Analyses were performed on the balanced sample which participated in all three waves of GUI98 and the three selected waves of analysis in GUI08, and for whom an interview date is available (GUI98 *N* = 5,986; GUI08 *N* = 7,507). To avoid uncertainty in the main variables of interest, the sample was further conditioned on having the same primary caregiver throughout the period of interviews, which resulted in a final sample of 5,748 for GUI98, and 7,208 for GUI08. A unique feature of the GUI data is that it covers a period of large economic fluctuation, with data collection for Wave 1 of GUI98 commencing during a period of near full employment during the Irish economic boom (2007/08), with subsequent data collection taking place during periods of high unemployment and economic hardship during the height of the Great Recession (2011/12), followed by a period of recovery as unemployment rates declined (2015/16). Data collection for Wave 2 of GUI08 took place just prior to the height of the economic recession (2010/11), with subsequent data collection time points taking place during a period of economic austerity (2013) and of economic recovery (2017/18) (Fig. 1).

### Variables

#### Internalized and externalized behavioral difficulties

The dependent variables were two dimensions of children’s behavioral problems: internalized and externalized problem behavior. Internalizing symptoms are those that primarily occur within the individual, such as emotional problems (e.g. anxiety, depression) and peer relationship problems (e.g. being solitary, playing alone). Externalizing symptoms reflect behavioral attributes that are externalized and visible to others (e.g. conduct difficulties and hyperactivity/inattention). These two dimensions were assessed using the Strengths and Difficulties Questionnaire (SDQ), which is a commonly used child mental health questionnaire consisting of 25 items in five subscales: emotional problems, peer problems, behavioral problems, hyperactivity and prosocial behavior (Goodman, 1997). While the use of all five subscales separately is advised when screening for disorder, the grouping of emotional and peer subscales into an ‘internalizing’ subscale, and conduct problems and hyperactivity into an ‘externalizing’ subscale have theoretical and empirical advantages when measuring internalizing and externalizing behavioral difficulty in low risk samples (Goodman et al., 2010). The internalizing and externalizing subscales have also been operationalized as such in a large body of literature on child behavioral difficulties (Flouri et al., 2015; Schenck-Fontaine & Panico, 2019).

The externalized behavior scale was created by adding the conduct and hyperactivity subscales, and the internalized behavior scale was constructed by adding the emotional and peer subscales. Cronbach’s alpha values for GUI98 internalizing behavior (Wave 1: 0.6928; Wave 2: 0.7094; Wave 3: 0.7184) and externalizing behavior (Wave 1: 0.7467; Wave 2: 0.7699; Wave 3: 0.7504) indicated acceptable internal consistency, with values for GUI08 outcomes of internalizing behavior (Wave 2: 0.5379; Wave 3: 0.6402; Wave 5: 0.7312) and externalizing behavior (Wave 2: 0.7314; Wave 3: 0.7633; Wave 5: 0.7867) likewise at acceptable levels. The resultant subscales could range from 0–20, with the original order reverse-coded so that a higher score implied greater behavioral difficulties.

#### Equivalized household income

To allow for comparisons between households that may differ in their composition in terms of size and age, we used the equivalized household income based on the nominal self-reported disposable household income (i.e., the self-reported total gross household income less statutory deductions of income tax and social insurance contributions) which was then equivalized using the Irish equivalence scale. On this scale every first adult carries a weight of 1, every additional adult (i.e., all aged 14 years or older) 0.66 and every child 0.33. The range of the variable in GUI98 was W1: 504-223116 (median: 19195), W2: 549-215849 (median 15849) and W3: 504-1214286 (median 14731). The range of the variable in GUI08 was W1: 1121 - 251256 (17069), W2: 2500-100000 (16081) and W3: 2590-322150 (18120). The distribution of the variable was right-skewed, and, hence, it was log-transformed.

#### Material deprivation

The material deprivation measure was a dummy, based on 11 items that are part of the official material deprivation indicator for Ireland (Central Statistics Office, 2021b) and that measures a household’s deprivation of food, clothing, heating and participation in family and social life: (1) Does your household eat meals with meat, chicken, fish (or vegetarian equivalent) at least every second day?; (2) Does your household have a roast joint (or its equivalent) at least once a week?; (3) Do household members buy new rather than second-hand clothes?; (4) Does each household member possess a warm waterproof coat?; (5) Does each household member possess two pairs of strong shoes?; (6) Does the household replace any worn out furniture?; (7) Does the household keep the home adequately warm?; (8) Does the household have family or friends for a drink or meal once a month?; (9) Does the household buy presents for family or friends at least once a year?; (10) Have you ever had to go without heating during the last 12 months through lack of money?; (11) Did you have a morning, afternoon or evening out in the last fortnight, for your entertainment (something that cost money)?. These items refer to socially perceived necessities in contemporary Ireland: inability to afford two or more of these counts as deprivation (Maître & Privalko, 2021). However, for the analysis a threshold of lacking one or more of these items was drawn to capture more people vulnerable to material deprivation. Findings are robust to alternative specifications of the deprivation variable.

#### Subjective financial strain

To assess the household’s subjective financial strain, responses to a question on the degree or ease with which the household could make ends meet were used. The answer categories ranged from 1 “with great difficulty” to 6 “very easily”, but the order was reverse-coded in the analysis so that a higher score indicated greater strain.

#### Household type

Finally, all analyses controlled for the type of household the study child lived in because the household type may change over time and affect the young person’s behavior. Household type was included as a categorical variable: (1) One-parent household with one or two children; (2) One-parent household with three or more children; (3) Two-parent household with one or two children; (4) Two-parent household with three or more children.

### Analytical Strategy

Analyses were performed in two steps. Firstly, descriptive results were presented and changes in the key measures over the course of the recession were explored. Secondly, longitudinal within-child fixed effects modeling was used to examine the relationship between the three indicators of economic well-being on the one hand, and internalized and externalized problem behavior on the other hand. For the fixed effects analyses, a stepwise approach was adopted in which the effect of each economic well-being variable was looked at separately before running a model that included all three simultaneously. Furthermore, research has identified differences in how boys and girls develop behavioral difficulties, with boys more likely to externalize their difficulties, while girls are more likely to internalize (Achenbach et al., 2002; Zahn-Waxler et al., 2008). However, because sex is a time-invariant variable, and fixed effects models control for all time-invariant heterogeneity, it could not be included as a predictor in the models. Thus, models were run separately for boys and girls. Non-response on analysis variables was generally low, but accounted for up to 9.5% of responses of the income question in GUI98 and 9.2% of responses to the income question in GUI08, the two variables with the highest proportion of missing responses in the datasets. To account for missing responses, all missing responses in model variables were imputed by performing multiple imputation with chained equations (MICE) with 25 iterations in STATA 17.0 (Sterne et al., 2009). The imputation model included all variables used in the main analysis as well as a set of auxiliary variables (Allison, 2012; Enders, 2010). Additionally, because non-random attrition might have introduced bias in the sample, analyses were weighted using the weighting factor provided in the GUI dataset for the balanced samples (Wave 3 for the GUI98; Wave 5 for the GUI08) to reflect population characteristics. The adoption of a multilevel models, such as a random effects or a mixed effects model, would allow for the estimation of both time-variant (e.g. changes within an individual over time) and time-invariant (e.g. level 2 variables such as class, sex and ethnicity) effects (Schunck, 2013). This is illustrated in the equation below (1)

1 $$Y_{it} = \beta _0 + \beta _1X_{it} + \mu _i + \varepsilon _{it}$$

where subscript *i* denotes variables that vary between individuals and *t* denotes variables that vary within individuals over time. As such, *X*_*it*_ is an independent variable that varies between and within individuals over time, *μ*_*i*_ is the unobserved heterogeneity (time invariant), and *ε*_*it*_ is the idiosyncratic error. However, accurate estimation of a multilevel model rests on the assumption that random intercepts are uncorrelated with the regressors. Where this assumption is violated, endogeneity bias will affect the accuracy of estimation (Antonakis et al., 2021). Fixed effects models hold several advantages over multilevel models because they remove all unobserved time constant heterogeneity from a model through differencing, subtracting the between model from the random effects model (2), thereby only leaving the fixed effects model without the random intercept (3).

2 $$\left( {Y_{it} - \bar Y_i} \right) = \beta _1\left( {X_{it} - \bar X_i} \right) + \left( {\mu _i - \bar \mu _i} \right) + \left( {\varepsilon _{it} - \bar \varepsilon _i} \right)$$

3 $$\tilde Y_{it} = \beta _1\tilde X_{it} + \tilde \varepsilon _{it}$$

By only comparing the same individual over time, fixed effect models control for all observed and unobserved variables that are time invariant (e.g., occupational class and ethnicity), and are therefore better able to account for confounders than many other models (Allison, 2009; Mummolo & Peterson, 2018). As this study is primarily interested in ascertaining the association between changes in economic well-being and child behavior net of time-invariant confounders, the adoption of a fixed effects model is suitable. However, a fixed effects model can only be run with variables that have enough within-variation over time. Whereas measures of economic well-being usually remain largely stable over time, the severity and suddenness of economic contraction during the period of GUI data collection increased variability in within-household experiences of economic well-being, thereby allowing us to measure within-variation while controlling for all between-characteristics through fixed effects analysis.

## Results

### Descriptive Results

The effects of the recession can be seen in the variation of mean levels of the three economic well-being variables across the three waves for all subsamples of GUI98 (see Table 1) and GUI08 (see Table 2). For GUI98, strain increases between Wave 1 and 2 before going down slightly between Wave 2 and 3, although it does not return to pre-recession levels. Similarly, there is a sharp increase in the proportion of households experiencing some type of material deprivation during the first years of the recession which only decreases moderately in the aftermath of the recession. The average household income drops substantially in the first years after the start of the recession and continues to decline in the later years albeit not as rapidly.

**Table 1:** GUI98: means and standard deviations for our key variables by sex

Boys	Wave 1		Wave 2		Wave 3	
	M	SD	M	SD	M	SD
Strain	2.93	1.09	3.70	1.17	3.49	1.24
Log income	9.76	0.50	9.57	0.52	9.49	0.56
Material deprivation	0.11	0.32	0.25	0.43	0.23	0.42
Internalized SDQ	3.21	3.00	2.88	2.93	2.91	2.79
Externalized SDQ	4.89	3.44	4.31	3.49	3.71	3.22

Girls	Wave 1		Wave 2		Wave 3	
	M	SD	M	SD	M	SD
Strain	2.99	1.12	3.80	1.17	3.57	1.21
Log income	9.68	0.58	9.52	0.55	9.42	0.56
Material deprivation	0.15	0.36	0.30	0.46	0.27	0.44
Internalized SDQ	3.50	2.99	3.20	2.95	3.94	3.16
Externalized SDQ	4.11	3.24	3.53	3.23	3.13	2.96

Weighted, unimputed data

**Table 2:** GUI08: means and standard deviations for our key variables by sex

Boys	Wave 2		Wave 3		Wave 5	
	M	SD	M	SD	M	SD
Strain	3.75	1.14	3.91	1.13	3.38	1.11
Log income	9.66	0.53	9.62	0.53	9.72	0.54
Material deprivation	0.31	0.46	0.35	0.48	0.23	0.42
Internalized SDQ	2.69	2.25	2.72	2.62	3.28	3.18
Externalized SDQ	5.72	3.41	5.38	3.62	4.99	3.73

Girls	Wave 2		Wave 3		Wave 5	
	M	SD	M	SD	M	SD
Strain	3.81	1.12	3.95	1.11	3.40	1.08
Log income	9.62	0.53	9.59	0.52	9.71	0.52
Material deprivation	0.31	0.46	0.35	0.48	0.23	0.42
Internalized SDQ	2.52	2.18	2.56	2.44	3.20	2.96
Externalized SDQ	5.20	3.34	4.50	3.22	3.77	3.14

Weighted, unimputed data

In the GUI08 cohort, strain increases from Wave 2 to 3, coinciding with the height of the economic recession, before declining to its lowest level in Wave 5 in the aftermath of the recession. This pattern is mirrored in changes in income and material deprivation, with income declining from Wave 2 to 3, before increasing at Wave 5 to its highest level, while material deprivation increases from Wave 2 to 3, before declining at Wave 5. Differences in internalized behavioral difficulties between boys and girls are small, while boys hold higher levels of externalized behavioral difficulties than girls at each time point.

In GUI98, differences in the average levels of internalized and externalized behavioral difficulties can be observed for boys and girls. On average, girls have higher levels of internalized behavioral difficulties than boys. Conversely, boys exhibit greater externalized behavioral difficulties than girls. Despite variability in individual trajectories, behavior seems to improve on average as the study children grow older with the means of externalized and internalized difficulties decreasing over time.

In GUI08, boys exhibit higher levels of both internalized and externalized behavioral difficulties than girls at all time points. However, the difference in internalized behavioral difficulties between boys and girls narrows over time, with the difference at its smallest at Wave 5.

Within-wave correlations are all in the expected directions in both GUI98 (Table 3) and GUI08 (Table 4). In both cohorts, higher household income is associated with fewer behavioral difficulties at each wave. Additionally, material deprivation and greater financial strain are related to increased internalized and externalized behavioral difficulties at each time point. Furthermore, the three economic well-being measures show statistically significant, albeit only moderately strong correlations. This suggests that these dimensions of economic well-being are indeed related but not necessarily the same.

**Table 3:** GUI98 within-wave correlations between the key variables by sex at each wave (male below diagonal; female above the diagonal)

	Strain	Log income	Material deprivation	Internalized SDQ	Externalized SDQ
Strain W1	1	−0.43***	0.33***	0.21***	0.19***
Log income W1	−0.45***	1	−0.24***	−0.15***	−0.12***
Material deprivation W1	0.29***	−0.15***	1	0.15***	0.11***
Internalized SDQ W1	0.20***	−0.11***	0.15***	1	0.36***
Externalized SDQ W1	0.18***	−0.11***	0.10***	0.34***	1
Strain W2	1	−0.47***	0.48***	0.19***	0.17***
Log income W2	−0.46***	1	−0.32***	−0.14***	−0.16***
Material deprivation W2	0.47***	−0.30***	1	0.14***	0.12***
Internalized SDQ W2	0.17***	−0.10***	0.15***	1	0.41***
Externalized SDQ W2	0.14***	−0.12***	0.10***	0.35***	1
Strain W3	1	−0.46***	0.49***	0.14***	0.13***
Log income W3	−0.47***	1	−0.34***	−0.11***	−0.12***
Material deprivation W3	0.48***	−0.32***	1	0.16***	0.12***
Internalized SDQ W3	0.14***	−0.11***	0.13***	1	0.43***
Externalized SDQ W3	0.11***	−0.09***	0.10***	0.35***	1

Weighted, unimputed data

***p < 0.001

**Table 4:** GUI08 within-wave correlations between the key variables by sex at each wave (male below diagonal; female above the diagonal)

	Strain	Log income	Material deprivation	Internalized SDQ	Externalized SDQ
Strain W1	1	−0.46***	0.46***	0.09***	0.13***
Log income W1	−0.48***	1	−0.35***	−0.12***	−0.16***
Material deprivation W1	0.50***	−0.37***	1	0.08***	0.09***
Internalized SDQ W1	0.13***	−0.14***	0.13***	1	0.31***
Externalized SDQ W1	0.13***	−0.14***	0.11***	0.32***	1
Strain W2	1	−0.42***	0.49***	0.09***	0.11***
Log income W2	−0.44***	1	−0.34***	−0.09***	−0.14***
Material deprivation W2	0.50***	−0.34***	1	0.12***	0.12***
Internalized SDQ W2	0.13***	−0.11***	0.14***	1	0.35***
Externalized SDQ W2	0.14***	−0.12***	0.14***	0.34***	1
Strain W3	1	−0.45***	0.45***	0.16***	0.12***
Log income W3	−0.47***	1	−0.33***	−0.09***	−0.11***
Material deprivation W3	0.45***	−0.33***	1	0.16***	0.12***
Internalized SDQ W3	0.17***	−0.12***	0.16***	1	0.42***
Externalized SDQ W3	0.13***	−0.12***	0.12***	0.45***	1

Weighted, unimputed data

***p < 0.001

### Multivariate Results

Fixed effects models are run separately using the GUI98 and GUI08 samples stratified by sex. For the GUI98 cohort, results are largely similar for boys and girls but only partly confirm our first hypothesis that economic well-being is related to increased behavioral difficulties (see Table 5). Against expectations, household income does not seem to be associated with the behavior of the young person. This is the case for both boys and girls, and even holds in the income-only model. Conversely, children who grow up in households with greater financial strain appear to have more externalized behavioral difficulties. Increased financial strain is predictive of increased externalized behavioral difficulties for boys and girls, even after adjusting for material deprivation and income. For material deprivation, distinct effects can be seen by sex. Material deprivation is not linked to any internalized behavioral difficulties for either boys or girls. However, it is associated with greater externalized behavioral difficulties for boys, although this is not the case for girls.

**Table 5:** Results from the fixed effects regression predicting internalized and externalized behavioral difficulties for boys and girls, GUI98

Boys	Internalized SDQ				Externalized SDQ			
	Model 1	Model 2	Model 3	Model 4	Model 1	Model 2	Model 3	Model 4
	B (SE)	B (SE)	B (SE)	B (SE)	B (SE)	B (SE)	B (SE)	B (SE)
Wave (baseline, Wave 1)								
*Wave 2*	−0.433***	−0.375***	−0.366***	−0.424***	−0.711***	−0.639***	−0.602***	−0.721***
	(0.087)	(0.079)	(0.079)	(0.088)	(0.086)	(0.076)	(0.080)	(0.087)
*Wave 3*	−0.417***	−0.377***	−0.368***	−0.402***	−1.277***	−1.232***	−1.206***	−1.281***
	(0.097)	(0.091)	(0.092)	(0.097)	(0.096)	(0.091)	(0.097)	(0.100)
Strain	0.083			0.09	0.154**			0.134*
	(0.052)			(0.052)	(0.055)			(0.057)
Material deprivation		0.045		−0.003		0.343**		0.267*
		(0.126)		(0.123)		(0.125)		(0.126)
Log income			0.017	0.069			−0.044	0.056
			(0.133)	(0.136)			(0.129)	(0.130)
Constant	2.485***	2.752***	2.595	1.796	4.382***	4.812***	5.322***	3.845**
	(0.332)	(0.266)	(1.360)	(1.456)	(0.310)	(0.262)	(1.287)	(1.348)
Observations	2792	2792	2792	2792	2792	2792	2792	2792

Girls	Internalized SDQ				Externalized SDQ			
	Model 1	Model 2	Model 3	Model 4	Model 1	Model 2	Model 3	Model 4
	B (SE)	B (SE)	B (SE)	B (SE)	B (SE)	B (SE)	B (SE)	B (SE)
Wave (baseline, Wave 1)								
*Wave 2*	−0.398***	−0.345***	−0.316***	−0.395***	−0.659***	−0.576***	−0.574***	−650***
	(0.086)	(0.077)	(0.078)	(0.088)	(0.084)	(0.079)	(0.083)	(0.088)
*Wave 3*	0.353***	0.389***	0.420***	0.370***	−1.035***	−0.976***	−0.974***	−1.021***
	(0.097)	(0.093)	(0.102)	(0.104)	(0.094)	(0.090)	(0.106)	(0.108)
Strain	0.092			0.089	0.103*			0.112*
	(0.052)			(0.053)	(0.050)			(0.054)
Material deprivation		0.14		0.102		0.004		−0.052
		(0.127)		(0.128)		(0.129)		(0.130)
Log income			0.05	0.101			0.007	0.048
			(0.142)	(0.142)			(0.177)	(0.177)
Constant	3.755***	4.017***	3.566**	2.771*	3.763***	4.093***	4.031*	3.284
	(0.280)	(0.239)	(1.355)	(1.374)	(0.263)	(0.198)	(1.704)	(1.752)
Observations	2956	2956	2956	2956	2956	2956	2956	2956

All analyses are performed on weighted and imputed data, and control for household type

*p < 0.05; **p < 0.01; ***p < 0.001

Contrary to the first hypothesis and in line with model results from the GUI98 cohort, fixed effects models run using the younger GUI08 cohort indicate that household income is not associated with behavioral difficulties (Table 6). However, in contrast to the GUI98 models where increased financial strain is found to be associated with increased externalized behavioral difficulties in both boys and girls, financial strain is not associated with either internalized or externalized behavioral difficulties among the younger age sample of GUI08. An increase in material deprivation is found to be associated with an increase in boys’ internalized behavioral difficulties, even after adjusting for income and financial strain. However, this association is not present for girls, nor for externalized behavioral difficulties.

**Table 6:** Results from the fixed effects regression predicting internalized and externalized behavioral difficulties for boys and girls, GUI08

Boys	Internalized SDQ				Externalized SDQ			
	Model 1	Model 2	Model 3	Model 4	Model 1	Model 2	Model 3	Model 4
	B (SE)	B (SE)	B (SE)	B (SE)	B (SE)	B (SE)	B (SE)	B (SE)
Wave (baseline, Wave 1)								
*Wave 2*	0.03	0.029	0.041	0.025	−0.361***	−0.370***	−0.372***	−0.370***
	(0.055)	(0.054)	(0.054)	(0.055)	(0.074)	(0.074)	(0.074)	(0.074)
*Wave 3*	0.620***	0.613***	0.594***	0.626***	−0.775***	−0.758***	−0.752***	−0.763***
	(0.076)	(0.073)	(0.074)	(0.076)	(0.080)	(0.080)	(0.080)	(0.080)
Strain	0.064			0.046	−0.022			−0.057
	(0.046)			(0.049)	(0.053)			(0.056)
Material deprivation		0.245*		0.226*		0.121		0.133
		(0.100)		(0.105)		(0.114)		(0.115)
Log income			0.026	0.077			−0.221	−0.238
			(0.113)	(0.113)			(0.127)	(0.135)
Constant	2.225***	2.386***	2.235*	1.462	5.373***	5.230***	7.411***	7.746***
	(0.392)	(0.316)	(1.083)	(1.145)	(0.408)	(0.351)	(1.240)	(1.407)
Observations	3620	3620	3620	3620	3620	3620	3620	3620

Girls	Internalized SDQ				Externalized SDQ			
	Model 1	Model 2	Model 3	Model 4	Model 1	Model 2	Model 3	Model 4
	B (SE)	B (SE)	B (SE)	B (SE)	B (SE)	B (SE)	B (SE)	B (SE)
Wave (baseline, Wave 1)								
*Wave 2*	0.042	0.043	0.046	0.042	−0.707***	−0.696***	−0.701***	−0.705***
	(0.056)	(0.056)	(0.055)	(0.056)	(0.072)	(0.072)	(0.072)	(0.072)
*Wave 3*	0.680***	0.677***	0.661***	0.672***	−1.408***	−1.439***	−1.424***	−1.410***
	(0.070)	(0.068)	(0.069)	(0.071)	(0.081)	(0.079)	(0.081)	(0.081)
Strain	0.013			0.023	0.055			0.065
	(0.041)			(0.042)	(0.049)			(0.050)
Material deprivation		0.026		0.025		−0.095		−0.133
		(0.103)		(0.104)		(0.115)		(0.116)
Log income			0.164	0.178			−0.085	−0.065
			(0.112)	(0.115)			(0.133)	(0.138)
Constant	2.206***	2.249***	0.687	0.462	4.680***	4.912***	5.701***	5.289***
	(0.355)	(0.324)	(1.156)	(1.226)	(0.388)	(0.362)	(1.406)	(1.494)
Observations	3588	3588	3588	3588	3588	3588	3588	3588

All analyses are performed on weighted and imputed data, and control for household type

*p < 0.05; **p < 0.01; ***p < 0.001

In line with the second hypothesis, results varied substantially by age cohort. However, while the anticipated association between financial strain and child behavioral difficulties among older children holds, the hypothesized relationship of resources with the development of behavioral difficulties in younger children only partly holds, with material deprivation associated with externalized behavioral difficulties for boys, and no observed effect for income on either internalized or externalized behavioral difficulties for either boys or girls.

### Sensitivity Analyses

The results from the main analysis provided only limited support for the expectation that the three economic well-being variables would be related to greater behavioral difficulties in early-to-middle childhood and adolescence. In order to confirm that the findings were robust to different operationalizations of the economic well-being variables and to check whether results remained stable when operationalizing variables in a similar manner to Schenck-Fontaine and Panico (2019), three additional models were run as robustness checks. In the first robustness check, material deprivation was measured as a continuous inversely weighted variable, so that infrequent experiences of material deprivation were given a greater weight than more common experiences of material deprivation. The second robustness check operationalized all economic well-being variables as dummies. The low income dummy distinguished between those who are 60% below the sample median and those who are above it, material deprivation was operationalized as in the main analyses and the financial strain dummy combined those who said they had difficulty or great difficulty making ends meet as opposed to all other categories. In the third robustness check, all economic well-being variables were again operationalized as dummies but based on different dichotomizations. This time, the financial strain dummy variable was operationalized such that any experience of strain was classified as experiencing financial strain, the alternative deprivation dummy was coded as in the main analyses, and the income dummy was based on the lowest income quintile.

Results remained largely stable to the different operationalizations of income, material deprivation and financial strain. Further details on the robustness checks employed and the results are available on request from the corresponding author.

## Discussion

A large body of literature has investigated the relationship between child behavior and economic well-being. However, the predominance of cross-sectional approaches and a focus on financial indicators has made it difficult to identify association over time and overlooked the potential heterogeneous effects of different aspects of economic well-being. This study sought to address some of these shortcomings by investigating the links between different experiences of family economic well-being and the development of internalized and externalized behavioral difficulties in two cohorts of young people between the ages of nine and 17 (GUI98) and three and nine years old (GUI08). The analysis covered a period of major economic fluctuation in Ireland: from just before the outbreak of the global financial and economic crisis (i.e. 2007/08) to the depths of the Irish recession (i.e. 2011-13) and, finally, economic recovery (i.e. year 2015 and after). This allowed us to disentangle the effect of economic well-being from time invariant confounders by measuring how changes in economic well-being within families (as opposed to between families), were associated with changes in children’s internalized and externalized behavioral difficulties. Furthermore, to develop a more comprehensive understanding of the heterogeneous effects of economic well-being, household economic well-being is conceptualized as multidimensional. This was done by using the available measures of disposable household income, material deprivation and financial strain to tap into the financial resources, living conditions and psychological financial stress aspects of economic well-being.

The effects of the recession were clearly visible in descriptive statistics of the key variables, with significant deterioration in economic well-being at the height of the recession. Moreover, in all waves, lower economic well-being in the three measures was associated with greater behavioral difficulties. However, fixed effects estimation provided only limited evidence for a relationship between income and child behavior in either of the cohorts. Meanwhile, an increase in household financial strain was related to greater externalized behavioral difficulties in boys and girls at later ages (GUI98) but had no association with either internalized or externalized behavioral difficulties in boys and girls at younger ages (GUI08), or internalized behavior at older ages (GUI98). Additionally, household material deprivation was linked to greater externalized behavioral difficulties in boys at later ages (GUI98) and internalized behavioral difficulties in boys at earlier ages (GUI08), but not found to be associated with internalized or externalized behavioral difficulties in girls in either age cohort. Interestingly, the results for income, material deprivation and financial strain remained even when adjusting for the other two economic well-being variables. Moreover, these findings were largely robust to different operationalizations of the income, material deprivation and strain variables. This suggests that each measure of economic well-being holds an independent effect that is not captured by either of the other two measures. This finding adds to the body of evidence on the importance of conceptualizing and measuring household economic well-being as multidimensional (Guio, 2018; Whelan et al., 2001), and is of policy interest, given it indicates that different experiences of economic well-being determine distinct child behavioral outcomes. While household income is a key measure of financial resources, it does not necessarily capture the material living conditions of families or the psychological aspects of coping with a given set of resources and needs. Hence, while household income remains an important and relevant indicator, it is important to consider it in combination with other aspects of household economic well-being when looking at child development. This has implications for theoretical models that incorporate household income, which may need to be adjusted to account for more multidimensional measures of economic well-being.

Placing the findings in the wider framework of economic well-being literature, the effects of material deprivation and financial strain corroborate the findings of existing studies. In line with FSM studies (Chzhen et al., 2021; Ponnet, 2014), a significant association was found between financial strain and externalized behavior outcomes among adolescent boys, net of other household characteristics. Besides, the finding that adolescent boys seem to be more likely to externalize difficulties is of particular relevance since a recent study among a large and representative sample of Swedish youth (Plenty et al., 2021) indicates that externalized behavioral difficulties for boys are associated with later-life likelihood of being Not in Employment, Education or Training (NEET). Similarly, the finding that material deprivation had an effect on younger boys’ internalized behavioral difficulties is of policy interest, given findings from previous research highlights the detrimental effect of economic hardship experienced in early ages on both short and long-term behavioral, cognitive and attainment outcomes (Duncan et al., 2010; Kiernan & Mensah, 2009).

However, the null finding regarding the effect of disposable household income on child behavior in either age cohort is somewhat surprising. While it is consistent with the finding of no significant income poverty association in fixed effects regression estimates with child behavior problems observed in recent research (Schenck-Fontaine & Panico, 2019), it contrasts with the body of observational evidence on the association between household income and child outcomes from the FSM literature. A potential explanation could be that the dependent variables do not capture the effect of changes in income on child outcomes, with prior research indicating that income poverty has a low association with emotional and behavioral development, but a stronger association with other child outcomes including cognition, educational attainment and self-rated health (Plenty & Mood, 2016). Furthermore, it could be that other factors, such as household debt, moderate the effect of financial resources, with income alone not able to accurately capture this effect (Conger et al., 1994). An alternative explanation is that income is associated with behavioral difficulties but is not the cause of them. This would explain why some studies (e.g. Schenck-Fontaine & Panico, 2019) observed income effects in models that rely on variation between households, but failed to observe the same effects when drawing on variation within households. Findings are also in line with the results of a recent systematic review of causal evidence of income effects on child outcomes (Cooper & Stewart, 2021), which documented limited evidence of direct household income effects on children’s behavior as opposed to via mediators such as parental mental health. This would suggest that there are other unobserved confounding factors that are captured by the income variable in between-household analyses, which also has implications for policy. Indeed, tax-benefit mechanisms can have a more direct effect on household incomes than on the actual living conditions or subjective evaluations of making ends meet.

Finally, this study holds several limitations, pointing at promising and relevant avenues for further research. First, the findings presented in this article might be specific to the Irish context and the Great Recession since Ireland experienced a unique and extreme change in economic climate. The possibility that these findings do not translate to different time periods, cohorts or countries cannot be dismissed, nor can it be categorically claimed that they hold in periods of milder economic decline. However, in the light of COVID-19 and the current cost-of-living crisis, these findings bear relevance for future studies. Second, by adopting fixed effects estimators, the models only look at change within families, and do not provide insight into whether the effect of the measures of economic well-being on child behavior differ by socio-economic status. It could be, for instance, that higher socio-economic households that experience income declines and increased financial strain are able to shield their children from the effects of such economic hardship owing to greater resources available to them prior to the onset of economic hardship, while change in income and financial strain among lower socio-economic households will more directly translate into effects on child behavior. The interaction between socio-economic background and economic well-being on child behavioral outcomes could be an interesting avenue for future research. However, given the primary interest of this article was to identify whether different experiences of economic well-being had independent effects on child behavioral outcomes, it was decided that it was not within the remit of this article to explore. Third, while fixed effects estimators, by virtue of the use of the individual as his/her own control, do not require a baseline value, the reliance on only within-individual variation over time discards all between-individual variation that could be used to estimate effects, leading to larger confidence intervals and less precise estimation when within-variance over time is small. It is therefore possible, particularly in the instance of Cohort08 where the sample timeline started later in the economic crisis, that effects which were present were not identified in the fixed effects models. Lastly, this study, while identifying different associations between measures of economic well-being and child behavioral outcomes, does not delve into the mechanisms through which these associations manifest themselves. Such an exploration is beyond the scope of this analysis – however, understanding how these different experiences of economic hardship lead to the development of varying child behavioral outcomes could prove an exciting avenue for future research.

## Conclusion

Much literature has sought to explore the effect of economic well-being on child behavioral outcomes. However, this research has struggled to ascertain to what degree observed associations are the result of direct links, or whether they reflect factors associated with economic well-being, such as social class and status. Drawing on unique Irish datasets that coincided with a period of major economic fluctuation in Ireland, this study utilized the variation in economic well-being variables to employ robust fixed effects analyses. This analysis controls for all time-invariant heterogeneity, thereby better isolating the effect of economic well-being on child behavioral outcomes net of time constant confounders. Household income was found not to be related to behavioral difficulties, whereas subjective financial strain was observed to be associated with externalized behavioral difficulties among older boys and girls. Material deprivation was associated with externalized behavioral difficulties in adolescent boys and internalized behavioral difficulties in younger boys, but not to have any effect on behavioral difficulties in girls at any age. These findings suggest that distinct experiences of household economic well-being have disparate effects on child behavioral difficulties.
